# Characterization of phenotypic spectrum of fetal heterotaxy syndrome by combining ultrasound and magnetic resonance imaging

**DOI:** 10.1002/uog.23705

**Published:** 2021-12-01

**Authors:** E. Seidl‐Mlczoch, G. Kasprian, A. Ba‐ssalamah, M. Stuempflen, E. Kitzmueller, D. A. Muin, D. Zimpfer, D. Prayer, I. Michel‐behnke, B. Ulm

**Affiliations:** ^1^ Pediatric Heart Center, Department of Pediatrics and Adolescent Medicine, Division of Pediatric Cardiology Medical University of Vienna Vienna Austria; ^2^ Department of Biomedical Imaging and Image‐guided Therapy, Division of Neuroradiology and Musculoskeletal Radiology Medical University of Vienna, Vienna Austria; ^3^ Department of Obstetrics and Gynaecology, Division of Obstetrics and Fetomaternal Medicine Medical University of Vienna, Vienna Austria; ^4^ Department of Cardiac Surgery, Pediatric Heart Center Vienna Medical University of Vienna, Vienna Austria

**Keywords:** fetal echocardiography, fetal MRI, heterotaxy, isomerism, ultrasound

## Abstract

**Objective:**

Heterotaxy or isomerism of the atrial appendages is a congenital disorder with variable presentation, associated with both cardiac and non‐cardiac anomalies, which may have a serious impact on fetal outcome. The aim of this exploratory study was to assess the value of fetal magnetic resonance imaging (MRI), as a complementary tool to ultrasound, for describing the morphological spectrum encountered in heterotaxy.

**Methods:**

This retrospective study included 27 fetuses that underwent fetal MRI following prenatal suspicion of heterotaxy on ultrasound from 1998 to 2019 in a tertiary referral center. Heterotaxy was classified as left atrial isomerism (LAI) or right atrial isomerism (RAI) based on fetal echocardiography (FE) examination. In addition to routine prenatal ultrasound, fetal MRI was offered routinely to enhance the diagnosis of non‐cardiac anomalies, which might have been missed on ultrasound. Prenatal findings on ultrasound, FE and MRI were reviewed systematically and compared with those of postnatal imaging and autopsy reports.

**Results:**

Twenty‐seven fetuses with heterotaxy and cardiovascular pathology, of which 19 (70%) had LAI and eight (30%) had RAI, were included. Seven (7/19 (37%)) fetuses with LAI had normal intracardiac anatomy, whereas all fetuses with RAI had a cardiac malformation. All 27 fetuses had non‐cardiac anomalies on fetal MRI, including situs and splenic anomalies. In 12/19 (63%) fetuses with LAI, a specific abnormal configuration of the liver was observed on MRI. In three fetuses, fetal MRI revealed signs of total anomalous pulmonary venous connection obstruction. An abnormal bronchial tree pattern was suspected on prenatal MRI in 6/19 (32%) fetuses with LAI and 3/8 (38%) fetuses with RAI.

**Conclusions:**

Visualization on MRI of non‐cardiac anomalies in fetuses with suspected heterotaxy is feasible and can assist the complex diagnosis of this condition, despite its limitations. This modality potentially enables differentiation of less severe cases from more complex ones, which may have a poorer prognosis. Fetal MRI can assist in prenatal counseling and planning postnatal management. © 2021 The Authors. *Ultrasound in Obstetrics & Gynecology* published by John Wiley & Sons Ltd on behalf of International Society of Ultrasound in Obstetrics and Gynecology.

**CONTRIBUTION**

*What are the novel findings of this work?*

In fetuses with a diagnosis of heterotaxy on ultrasound, fetal magnetic resonance imaging (MRI) was helpful in the identification of abdominal situs anomalies, malrotation of the intestines, splenic conditions and other non‐cardiac structural anomalies. We also found subtype‐specific bronchial patterns in fetuses with right atrial isomerism and those with left atrial isomerism (LAI) using MRI. A specific configuration of the fetal liver, characterized by the presence of a ‘hook‐shaped’ portion of liver parenchyma, was identified on MRI in fetuses with LAI.

*What are the clinical implications of this work?*

Fetal MRI can be helpful in the classification of heterotaxy, as non‐cardiac anomalies were detected systematically on MRI. These findings may further facilitate prenatal counseling and postnatal management of heterotaxy.

## INTRODUCTION

Heterotaxy or isomerism of the atrial appendages is a rare congenital disorder with an incidence of 1 in 5000–7000 live births^1^, characterized by an unspecific abnormal arrangement of the left–right axis of the fetal body^2^, ^3^. Left atrial isomerism (LAI) is distinguished from right atrial isomerism (RAI) by pathognomonic patterns. In LAI, there are typically two left atrial appendages (defined by the specific muscle pattern)^4^ and a situs anomaly of the abdominal organs, typically with a balanced congenital heart defect (CHD), an interrupted inferior vena cava and non‐cardiac conditions such as malrotation of the intestines and polysplenia. In RAI, two right atrial appendages can be found, together with a severely unbalanced CHD, involving abnormal drainage of the pulmonary veins and abnormal intra‐abdominal location of the aorta and inferior vena cava, and asplenia in the majority of cases^2^, ^3^. Discordance between bronchopulmonary branching, arrangement of the atrial appendages and splenic status in more than one‐fifth of patients with heterotaxy has been reported^5^. Both nomenclature and classification of heterotaxy are matters for discussion; therefore, the International Society for Nomenclature of Paediatric and Congenital Heart Disease proposed a definition in 2007^6^ to facilitate the precise use of the term, which we use in this study.

The variability of heterotaxy presentation challenges fetal imaging interpretation. Reported investigations on fetal heterotaxy rely mainly on ultrasound examination, including fetal echocardiography (FE), and autopsy findings^7^, ^8^, ^9^, ^10^, ^11^, ^12^, ^13^. In our center, fetal magnetic resonance imaging (MRI) is offered routinely to all patients with suspicion of severe non‐cardiac anomalies on ultrasound. Fetal MRI complements ultrasound by providing unique tissue contrast and characterization^14^.

As technical and maternal limitations (e.g. maternal obesity, fetal position, lack of amniotic fluid and those related to ultrasound machines) influence the detection of cardiac and non‐cardiac anomalies on prenatal ultrasound, fetal MRI can be used as an adjunct to ultrasound and FE to facilitate prenatal characterization of the condition and counseling regarding postnatal interventional strategies. To date, there have been a limited number of studies investigating heterotaxy using fetal MRI, with small numbers of cases included^2^, ^15^, ^16^.

The aim of this study was to assess the additional value of fetal MRI in the characterization of heterotaxy *in utero*, but a comparison of different imaging modalities was not one of the objectives of the study.

## METHODS

This retrospective series included MRI scans of 27 fetuses with a diagnosis of fetal heterotaxy between January 1998 and December 2019. Patients were identified from fetal cardiac databases of the Department for Obstetrics and Gynecology, Division of Pediatric Cardiology and Department of Pediatric and Adolescent Medicine of the Medical University of Vienna. The ethics committee of the Medical University of Vienna approved the study protocol (1306/2020). During the study period, mid‐trimester fetal ultrasound scans were carried out in a standardized fashion according to the International Society of Ultrasound in Obstetrics and Gynecology (ISUOG) guidelines^17^. Following the ultrasound examination that raised suspicion of heterotaxy syndrome, detailed FE was performed. The type of heterotaxy was determined on FE in the abdominal situs view. The presence of an interrupted inferior vena cava with azygos continuation was considered indicative of left isomerism, while juxtaposition of the aorta and inferior vena cava in combination with a cardiac malformation and abnormal cardiac or abdominal organ situs were considered to be markers of right isomerism^18^, ^19^, ^20^. Following the prenatal diagnosis of heterotaxy, all patients were offered genetic assessment and fetal MRI for further evaluation. A retrospective chart review of available prenatal and postnatal ultrasound reports and medical records (autopsy reports) was performed. Available fetal MRI images were evaluated systematically, with a focus on heterotaxy‐specific findings.

**Table 1:** Clinical characteristics of study population (*n* = 27)

Characteristic	Value
Maternal age at diagnosis (years)	31 (20–42)
Gestational age at diagnosis (weeks)	23 (14–37)
Gestational age at fetal MRI (weeks)	25 (17–38)
Gender known	25 (93)
Male sex	12/25 (48)
Genetic assessment performed	18 (67)
LAI	19 (70)
SUA	4/19 (21)
Nuchal translucency thickness > 2.5 mm	0 (0)
Growth restriction	1/19 (5)
Termination of pregnancy	2/19 (11)
RAI	8 (30)
SUA	2/8 (25)
Nuchal translucency thickness > 2.5 mm	1/8 (13)
Growth restriction	0 (0)
Termination of pregnancy	2/8 (25)

Data are given as mean (range), *n* (%) or *n*/*N* (%).

LAI, left atrial isomerism; MRI, magnetic resonance imaging; RAI, right atrial isomerism; SUA, single umbilical artery.

**Table 2:** Cardiovascular malformations in 27 fetuses with heterotaxy detected on fetal and postnatal echocardiography, according to whether they had left atrial isomerism (LAI) or right atrial isomerism (RAI)

Finding	LAI (*n* = 19)	RAI (*n* = 8)
Systemic venous anomaly		
Bilateral superior vena cava	6 (32)	3 (38)
Pulmonary venous anomaly		
Total anomalous pulmonary venous connection	1 (5)	4 (50)
Partial anomalous pulmonary venous connection	4 (21)	2 (25)
Septation defect		
Isolated ventricular septal defect	1 (5)	0
Common atrium	1 (5)	2 (25)
Complete atrioventricular septal defect	3 (16)	4 (50)
Anomaly of the ventricles		
Hypoplastic left ventricle/single right ventricle	3 (16)	2 (25)
Hypoplastic right ventricle/single left ventricle	2 (11)	1 (13)
Single‐ventricle morphology	2 (11)	3 (38)
Double‐chambered right ventricle	1 (5)	0
Ventriculoarterial anomaly		
D‐transposition/malposition of great arteries	3 (16)	3 (38)
Double‐outlet right ventricle	3 (16)	2 (25)
Double‐outlet left ventricle	1 (5)	0
Truncus arteriosus communis	0	1 (13)
Outflow tract obstruction		
Pulmonary stenosis/atresia	5 (26)	6 (75)
Aortic stenosis/atresia	2 (11)	1 (13)
Coarctation of the aorta	4 (21)	0
Heart rhythm		
Sinus rhythm	14 (74)	8 (100)
Higher‐degree heart block	2 (11)	0
Sinus rhythm, postnatal sinus bradycardia	2 (11)	1 (13)
Sinus rhythm, postnatal heart block	1 (5)	0

Data are given as *n* (%).

Some fetuses had more than one cardiac anomaly.

Fully informed, written consent was obtained from all parents prior to fetal ultrasound, MRI, genetic investigation, postnatal imaging and autopsy. Prenatal ultrasound examination and FE were performed on a variety of ultrasound machines during the study period: GE Voluson E8/E10 (GE Healthcare, Zipf, Austria), GE Vivid 7, Vivid E9, GE LOGIQ (GE Healthcare) and Philips EPIQ 7 (Philips Healthcare, Austria).

### Fetal MRI

Fetal MRI was performed without maternal sedation using a 1.5‐Tesla MR system, an 8‐channel body coil and a protocol that followed ISUOG guidelines^21^, using ultrafast T2‐weighted sequences, performed in three orthogonal planes, T1‐weighted gradient‐echo, diffusion‐weighted and steady‐state free precession sequences. An expert in fetal MRI (G.K.) with 15 years of experience performed a standardized analysis of all MRI data in a systematic fashion. The following features were addressed with a specific focus on their relationship to the midline of the fetal body: the position of the cardiac apex, position and size of the large cardiac vessels, umbilical vein, position and size of the stomach, rotational appearance of the small and large intestines, shape and configuration of the fetal liver and presence or absence of spleen tissue. In addition, the feasibility of characterizing the branching pattern of the bronchial tree and the appearance of the pulmonary lobes was evaluated. The gold standard postnatal definition of bilateral hyparterial or eparterial bronchus was difficult to use in the setting of fetal MRI; therefore, the following definition was adopted: bronchial tree pattern with short bronchial arms and an early bilateral rise of the upper lobe bronchi with a short distance to the middle lobe bronchi was considered as typical of RAI, while a bronchial tree pattern with long bronchial arms and a late bilateral rise of the upper lobe bronchi was considered as typical of LAI^2^.

The ‘nutmeg’ lung pattern was defined on T2‐weighted images as a heterogeneous signal, with tubular structures radiating peripherally from the hila, indicating pulmonary lymphangiectasia.

### Statistical analysis

Only descriptive statistics were applied. Demographic, anatomical and outcome data were included in the analysis. Data are presented as *n* (%), *n*/*N* (%), mean (range) or median (range), as appropriate. Postnatal findings (when available) were compared with fetal findings.

## RESULTS

**Figure 1:**
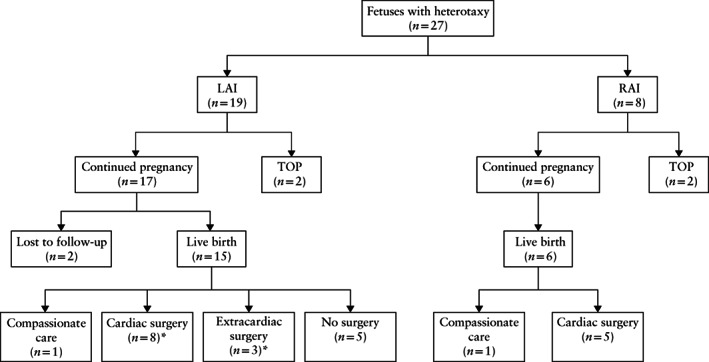
Flowchart of fetuses with heterotaxy that underwent detailed fetal magnetic resonance imaging evaluation and were included in the analysis. *Two fetuses had both cardiac surgery and extracardiac surgery. LAI, left atrial isomerism; RAI, right atrial isomerism; TOP, termination of pregnancy.

Twenty‐seven fetuses had a prenatal diagnosis of heterotaxy and underwent further evaluation on fetal MRI, meeting the inclusion criteria of the study (Figure 1). Nineteen (70%) fetuses were categorized as LAI and eight (30%) were categorized as RAI based on FE findings. Clinical characteristics of the study population are shown in Table 1. Termination of pregnancy occurred in 2/19 (11%) fetuses with LAI and 2/8 (25%) fetuses with RAI. Overall, 15/19 (79%) fetuses with LAI and 6/8 (75%) fetuses with RAI were liveborn (two fetuses with LAI were lost to follow‐up during pregnancy) (Figure 1). Mean gestational age at diagnosis was 23 weeks (range, 14–37 weeks). Fetal MRI was performed at a mean gestational age of 25 weeks (range, 17–38 weeks) (Table 1).

Cardiovascular malformations according to the type of isomerism are listed in Table 2. Seven (37%) fetuses with LAI had normal intracardiac anatomy, whereas all fetuses with RAI had a cardiac malformation. Delayed or interrupted conduction between the atria and ventricles was defined as atrioventricular block. Higher‐degree heart block was detected in 2/19 (11%) fetuses with LAI and was not present in fetuses with RAI. Neither of the two fetuses with higher‐degree heart block developed hydrops. Complex intracardiac malformation with obstruction of the right ventricular outflow tract was more common in fetuses with RAI (6/8 (75%)) than in those with LAI (5/19 (26%)). Complete atrioventricular septal defects were more common in fetuses with RAI (4/8 (50%)) than in those with LAI (3/19 (16%)). Coarctation of the aorta was present only in fetuses with LAI (4/19 (21%)). D‐transposition of the great arteries was seen in 3/8 (38%) fetuses with RAI and 3/19 (16%) fetuses with LAI. Hypoplastic left ventricle or single right ventricle was found in 3/19 (16%) fetuses with LAI and 2/8 (25%) fetuses with RAI. Single‐ventricle morphology was more common in cases with RAI (3/8 (38%)) than in those with LAI (2/19 (11%)). Fetal genetic testing was performed in 67% (18/27) of all fetuses. All fetuses had a basic chromosomal investigation using karyotyping; one fetus was tested using microarray and one using non‐invasive prenatal testing. One fetus was diagnosed with 22q11 microdeletion and another with Bardet–Biedel syndrome. One child tested positive for Jeune syndrome (*ATD3*) postnatally.

### MRI findings

**Figure 2:**
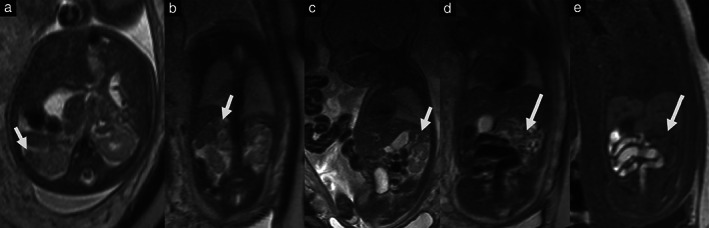
Magnetic resonance images in a fetus with left atrial isomerism at 28 + 6 weeks' gestation, showing polysplenia (short arrow) (a–c) and intestinal malrotation, demonstrated by absence of colonic flexure in left epigastric region (long arrow) (d,e).

**Figure 3:**
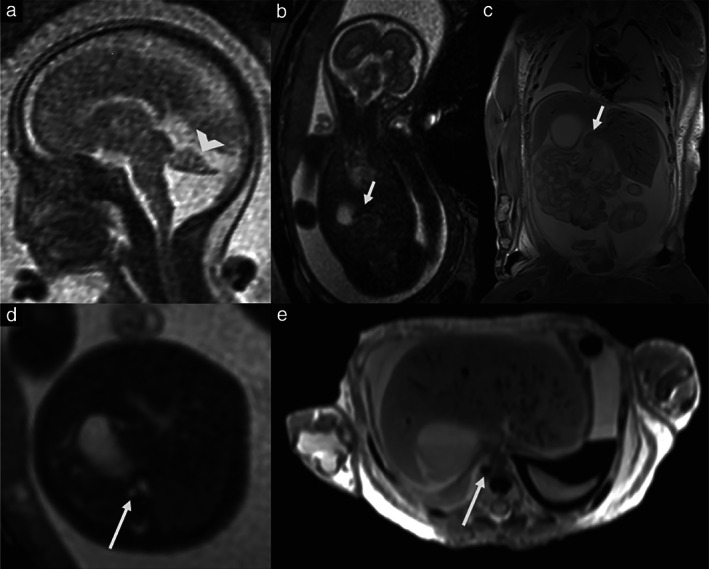
Magnetic resonance images in a fetus with left atrial isomerism, obtained at 23 + 0 weeks' gestation (a,b,d) and postmortem at 23 + 4 weeks (c,e), showing Dandy–Walker malformation with vermian hypoplasia and intestinal malrotation (arrowhead) (a), abnormal liver configuration with an additional liver lobule (short arrow) (b,c) and an azygos vein with interruption of the inferior vena cava (long arrow) (d,e).

Two fetuses with LAI underwent two MRI scans, resulting in a total of 29 MRI scans performed at a mean gestational age of 25 weeks (range, 17–38 weeks). Fetal MRI findings with postnatal confirmation are summarized in Table 3. Overall, 63% of fetuses had one or more non‐cardiac anomalies on fetal MRI. Examples of specific fetal MRI findings are shown in Figures 2 and 3.

**Table 3:** Fetal magnetic resonance imaging (MRI) findings with postnatal confirmation in 27 fetuses with heterotaxy, according to whether they had left atrial isomerism (LAI) or right atrial isomerism (RAI)

Finding	LAI (*n* = 19)	RAI (*n* = 8)
Situs		
Anomaly	17 (89)	6 (75)
Inversus	2 (11)	2 (25)
Stomach position		
Midline	0	1 (13)
Right	18 (95)	5 (63)
Left	1 (5)	2 (25)
Heart/stomach position		
Ipsilateral	6 (32)	0
Contralateral	13 (68)	8 (100)
Dextrocardia	5 (26)	2 (25)
Brain		
Ventricular asymmetry	1 (5)	1 (13)
Dandy–Walker malformation	1 (5)	0
Cerebellar hypoplasia	0	1 (13)
Hydrocephalus	0	1 (13)
Stenosis of the aqueduct	0	1 (13)
Ventriculomegaly	0	1 (13)
Craniofacial		
Hypertelorism	1 (5)	0
Cleft lip/palate	1 (5)	0
Retrognathia	0	1 (13)
Facial dysmorphism	1 (5)	0
Skeletal		
Short curved femur	1 (5)	0
Lungs		
Suspected abnormal branching pattern	6 (32)	3 (38)
‘Nutmeg’ pattern	1 (5)	2 (25)
Gastrointestinal tract		
Liver		
Midline	17 (89)	6 (75)
Left	2 (11)	2 (25)
Abnormal liver configuration	12 (63)	0
Gallbladder		
Midline	4 (21)	3 (38)
Left	2 (11)	1 (13)
Gallbladder aplasia	1 (5)	0
Malrotation of the intestines	6 (32)	3 (38)
Esophageal atresia	0	1 (13)*
Duodenal atresia	2 (11)	0
Spleen		
Asplenia	4 (21)	6 (75)
Polysplenia	2 (11)	0
Right‐sided	4 (21)	2 (25)
Abnormally small	1 (5)	0
Urinary tract		
Duplex kidney (unilateral/bilateral)	1 (5)	1 (13)
Unilateral kidney agenesis	0	1 (13)
Polycystic kidneys	1 (5)	0
Umbilical vein		
Abnormal course	6 (32)	1 (13)
Persistent right umbilical vein	1 (5)	0

Data are given as *n* (%).

Some fetuses had more than one non‐cardiac anomaly.

* Found postnatally to have duodenal atresia.

Five fetuses, including 2/19 (11%) fetuses with LAI and 3/8 (38%) fetuses with RAI, had abnormal central nervous system findings on MRI. One of these fetuses with LAI had Dandy–Walker malformation, while the other had ventricular asymmetry. Of the eight fetuses with RAI, one (13%) had cerebellar hypoplasia, hydrocephalus and stenosis of the aqueduct, one (13%) had ventricular asymmetry and one (13%) had ventriculomegaly. In one other case with RAI, hydrocephalus was diagnosed in childhood.

Cleft lip and palate and hypertelorism were observed in one (5%) fetus with LAI. In addition, this fetus had a short, curved femur visible on fetal MRI. Retrognathia was diagnosed in 1/8 (13%) fetus with RAI, and facial dysmorphism was described in 1/19 (5%) fetus with LAI.

**Figure 4:**
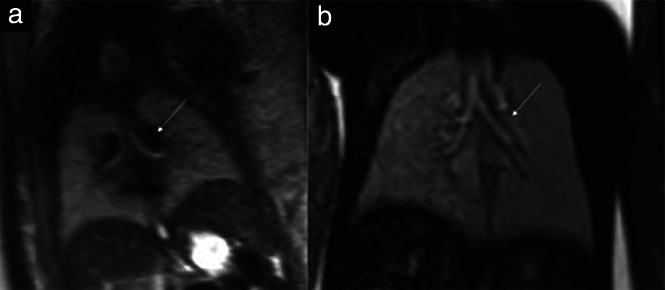
Magnetic resonance images of the pulmonary system in two fetuses with heterotaxy. (a) Fetus with right atrial isomerism at 25 + 5 weeks' gestation, presenting with abnormal branching pattern of the bronchial tree, with short bronchial arms (arrow). (b) Fetus with left atrial isomerism at 24 + 2 weeks, presenting with abnormal branching pattern of the bronchial tree, with long bronchial arms (arrow).

The fetal bronchial tree was characteristic of the subtype of heterotaxy in 9/27 (33%) fetuses, with double left‐sided bronchial pattern in 6/19 (32%) cases with LAI and double right‐sided bronchial pattern in 3/8 (38%) cases with RAI. The diagnosis was based on the shape and pattern of the main bronchial arms, as the rise of the upper lobe bronchi could not be seen clearly (Figure 4).

**Figure 5:**
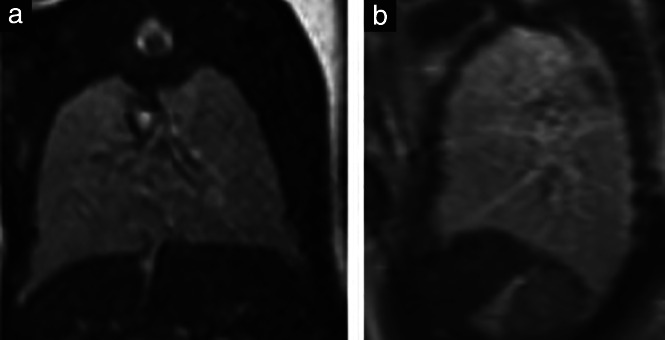
Coronal (a) and sagittal (b) T2‐weighted magnetic resonance images in a fetus with right atrial isomerism at 32 + 2 weeks' gestation. The fetal lungs had hyperintense linear signal intensity changes, with the characteristic nutmeg pattern of the lung, indicative of pulmonary lymphangiectasia, in the setting of obstructed total anomalous pulmonary venous drainage and heterotaxy.

Five (19%) fetuses, including one (5%) LAI case and four (50%) RAI cases, with total anomalous pulmonary venous connection (TAPVC) underwent fetal MRI at 22–37 weeks' gestation. Of the fetuses with TAPVC, 2/8 (25%) with RAI and 1/19 (5%) with LAI had pulmonary lymphangiectasia on MRI, which can be associated with TAPVC obstruction (Figure 5). The obstruction was suspected in two of those fetuses on FE. No pulmonary anomaly was seen on ultrasound. Postnatally, obstructed TAPVC was confirmed on transthoracic echocardiography and pulmonary lymphangiectasia was documented on chest X‐ray in two newborns. The other pregnancy with suspected obstruction of TAPVC on MRI was terminated, and no specific description of the lung was noted in the autopsy report.

In the other two fetuses with TAPVC and RAI, no sign of obstruction was detected on FE and the lungs appeared normal on fetal MRI. Postnatally, neither echocardiography nor chest X‐ray revealed TAPVC obstruction or pulmonary lymphangiectasia in these two cases. The lobulation pattern of the lungs could not be differentiated fully on fetal MRI in any of the cases.

Anomaly of the gastrointestinal organs was detected on MRI in all 27 fetuses. Fetal MRI revealed situs anomaly with a midline liver and atypical location of abdominal organs in 85% (23/27) of cases and situs inversus abdominalis in 15% (4/27) fetuses. A right‐sided stomach was seen in 18/19 (95%) fetuses with LAI and 5/8 (63%) fetuses with RAI. The heart/stomach position was contralateral in 13/19 (68%) fetuses with LAI and in all eight fetuses with RAI. Dextrocardia was documented in 5/19 (26%) fetuses with LAI and 2/8 (25%) fetuses with RAI.

**Figure 6:**
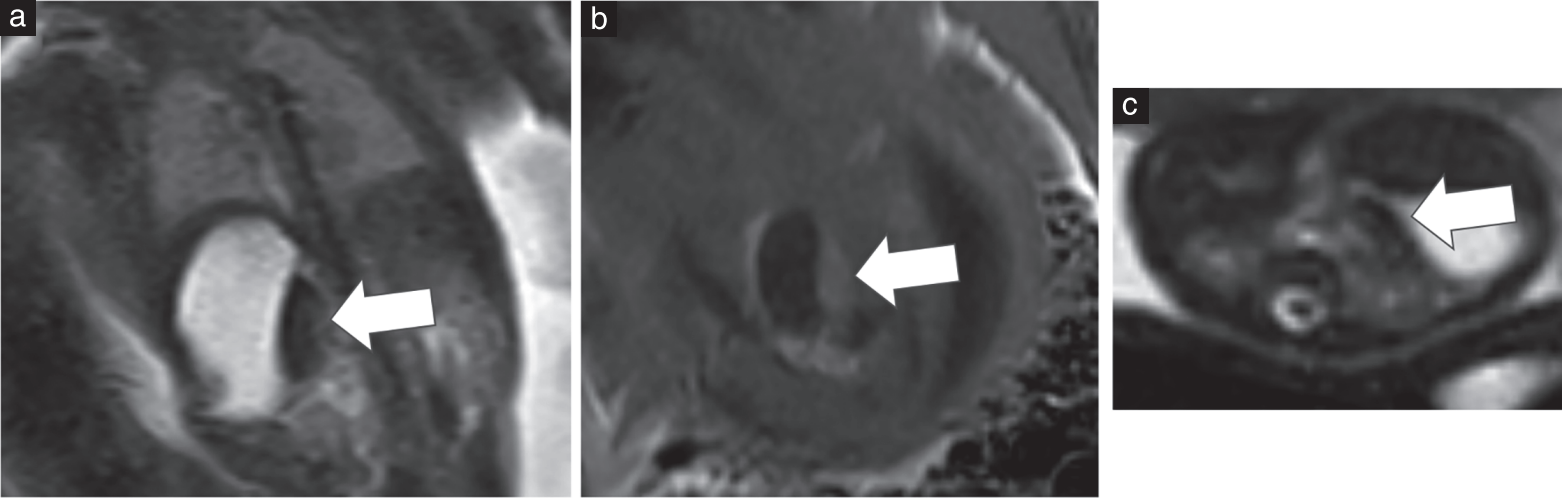
Coronal (a,b) and axial (c) magnetic resonance images of the fetal liver. ‘Hook‐shaped’ portion of liver parenchyma derived from left liver lobe appeared hypointense on T2‐weighted imaging (arrow) (a,c) and hyperintense on T1‐weighted imaging (arrow) (b). The abnormal liver segment extended towards the small curvature of the stomach.

A specific configuration of the fetal liver characterized by the presence of a ‘hook‐shaped’ portion of liver parenchyma was seen only in fetuses with LAI (12/19 (63%)) (Figure 6).

Malrotation of the intestines was noted on MRI in 9/27 (33%) fetuses, including six (32%) cases with LAI and three (38%) cases with RAI, and a further 2/27 (7%) cases were diagnosed on postnatal imaging.

Gallbladder aplasia was diagnosed in one fetus at 33 + 2 weeks' gestation on MRI, with biliary atresia and LAI confirmed postnatally. Esophageal atresia was suspected on MRI in one fetus with RAI, with a postnatal diagnosis of duodenal atresia. This fetus did not have the typical double‐bubble sign on ultrasound and the stomach was not filled at the time of fetal MRI (Table S1).

Diagnosis of splenic anomalies on fetal MRI was correct in 19/27 (70%) fetuses, including 11/19 (58%) fetuses with LAI (four fetuses with asplenia, two with polysplenia, one with an abnormally small spleen and four with a right‐sided spleen) and all eight cases with RAI (six fetuses with asplenia and two with a right‐sided spleen). All findings were confirmed by postnatal ultrasound examination or autopsy studies.

An abnormality of the kidneys was noted in two fetuses with LAI, including polycystic kidneys in one fetus (5%) and duplex kidney in another (5%). In the group with RAI, kidney agenesis was present in one fetus (13%) and bilateral duplex kidney in one other (13%).

Apart from the situs and anomalies of the spleen, fetal MRI revealed one or more non‐cardiac structural malformation(s) in 14/27 (52%) fetuses. Postnatal extracardiac findings such as uterine agenesis or biliary atresia are usually not detected on fetal MRI. However, biliary atresia was suspected owing to gallbladder aplasia in one case with LAI. All findings from postnatal imaging and autopsy studies that were missed on fetal MRI are listed in Table S1.

## DISCUSSION

This report on fetal MRI findings in 27 fetuses with heterotaxy is the largest fetal MRI study in this patient population to date. In the present study, fetal MRI documented information on abnormal arrangement of the internal organs as well as extracardiac anomalies (ECA) and helped to provide phenotypic characterization of fetal heterotaxy. Smaller fetal MRI series provided promising preliminary data on its ability to complement the diagnosis of heterotaxy^15^, ^16^. The definition of heterotaxy implies an abnormal arrangement of the abdominal organs, not always in a distinct pattern. The position of the liver in heterotaxy syndrome is especially variable. Both in RAI and in LAI, the liver may be positioned in the midline or predominantly in the right upper abdominal quadrant. Furthermore, a liver positioned predominantly in the left upper abdominal quadrant has been reported in both conditions^22^. In LAI, the left liver lobe has occasionally been described as being very prominent^23^. Here, we describe a specific configuration of the fetal liver encountered in LAI: a hook‐shaped portion of liver parenchyma, extending towards the small curvature of the stomach (Figure 6), derived apparently from the left liver lobe, either from liver segments 2/3 or 4a/b. Embryologically, the human liver bud is asymmetrical from its first appearance^24^. In animal models, it has recently been shown that lateralized expression of *Pitx2* results in cellular asymmetry of the epithelium of the hepatic diverticulum, leading to the generally asymmetrical liver morphogenesis^25^. Here, we were able to demonstrate an atypical liver morphology in association with a specific lateralization defect (LAI), which may be linked to an abnormal local expression of left lateralizing morphogens. Interestingly, the characteristic hook‐shaped liver configuration was not encountered in RAI cases. Previously, the classification of heterotaxy was based on the presence of asplenia or polysplenia^2^, ^3^, ^26^. Gaur *et al*.^16^ investigated 13 fetuses with polysplenia using fetal MRI and were able to visualize the spleen in 6/13 (46%) fetuses. In our study, fetal MRI diagnosis of the spleen was correct in 100% of RAI cases and 58% of LAI cases. The limitations of the diagnosis of the spleen lie in the smallness of the organ, especially at early fetal MRI examinations owing to low spatial resolution and fetal motion.

Examination of the bronchial‐tree pattern has been used in postnatal diagnostics^27^. The shape of the bronchial tree was typical for the heterotaxy subtype in 6/19 (32%) fetuses with LAI and 3/8 (38%) fetuses with RAI, which added clues to the characterization of heterotaxy.

A recent study by Yim *et al*.^5^ stated that the typical signs of isomerism were breached in over 20% of cases with heterotaxy. In 7.5% of investigated cases, there was a discrepancy between the arrangement of the bronchial‐tree pattern and that of the atrial appendages^5^.

In our study, we found concordance between the prenatally diagnosed subtype (LAI or RAI) and the bronchial‐tree pattern on postnatal imaging in four of the six cases investigated postnatally on computed tomography or MRI of the thorax.

ECAs may be present in fetuses with heterotaxy^26^, ^28^, ^29^, ^30^, regardless of the subtype. In our study, ECAs were present in 17/27 (63%) cases. Escobar‐Diaz *et al*.^29^ reported non‐cardiac anomalies in 62.2% of cases in a cohort of 154 fetuses with heterotaxy syndrome, while Gottschalk *et al*.^30^ reported a lower incidence of 15.8%. Ticho *et al*.^28^ emphasized the idea that the midline plays an important role in the formation of normal left–right asymmetry^30^, ^31^, ^32^, ^33^. Malrotation of the intestines was seen in 32% of LAI and 38% of RAI cases, with an overall detection rate of 82% (9/11) in our study. MRI helped to visualize meconium, which is difficult on ultrasound. Ticho *et al*.^28^ reported the presence of malrotation in 33% of their cohort.

Obstruction of TAPVC is a major risk factor for postnatal mortality. A study by Ganesan *et al*.^34^ investigated prenatal sonographic features of fetal TAPVC and found Doppler‐flow waveforms to be valuable in determining specific subtypes of TAPVC. Owing to the retrospective fashion of our study, it was not possible to evaluate Doppler waveforms in our TAPVC fetuses.

In our study, fetal MRI aided in identifying pulmonary features reflective of severely obstructed TAPVC. Three fetuses with suspected obstruction due to TAPVC on FE had a pulmonary nutmeg pattern sign on fetal MRI. The nutmeg pattern has been described in fetuses with hypoplastic left heart syndrome and restricted atrial septal flow^35^, and interpreted as a sign of lymphangiectasia and venous arterialization.

In our institution, fetal MRI is offered to all women with a cardiac and/or non‐cardiac anomaly on a voluntary basis. Prior to the examination, we make it clear that MRI does not guarantee additional diagnostic information to that provided by ultrasound evaluation.

This retrospective study has several limitations. Owing to its retrospective nature, the study was not a comparative study of ultrasound and fetal MRI. We included only fetuses with a fetal diagnosis of heterotaxy that underwent fetal MRI. Thus, ours was a highly selected patient cohort, and no general conclusions on sensitivity and specificity of the detection by MRI can be drawn from this study. It is beyond the scope of this study to state whether there are possible advantages of MRI over ultrasound, as these were not evaluated systematically. This study only provides information on the possibilities of MRI but does not imply a general recommendation for the use of this modality in the setting of heterotaxy. However, it might be of help as a complementary tool, if available. We are fully aware that the disharmony of arrangements of different organ systems challenges fetal and postnatal subtype specification, thus prenatal diagnosis of the subtype of left and right isomerism can never be fully complete. Postnatal evaluation is warranted to address this important question and learn more about disharmonious patterns of heterotaxy.

In conclusion, we have performed a systematic phenotypic characterization of fetuses with heterotaxy using widely available prenatal imaging modalities (MRI and ultrasound). We were able to identify a series of extracardiac abnormalities that could help to specify further the subtype of heterotaxy. Future prospective studies are needed to demonstrate if ultrasound is equivalent to MRI in the detection and/or exclusion of these findings in individual cases.

## Supporting information


**Table S1** Summary of non‐cardiac findings on fetal magnetic resonance imaging (MRI) and additional postnatal or autopsy findings in patients with prenatal diagnosis of heterotaxy, sorted by left atrial isomerism (LAI; *n* = 19) or right atrial isomerism (RAI; *n* = 8), according to gestational week of fetal MRI investigation

## Data Availability

Data available on request due to privacy/ethical restrictions
