# Supplementary material for: Characterization of phenotypic spectrum of fetal heterotaxy syndrome by combining ultrasound and magnetic resonance imaging
Source: Ultrasound Obstet Gynecol. 2021 Dec 1;58(6):837–45. doi: 10.1002/uog.23705 (PMC9299896; doi:10.1002/uog.23705)
Supplement: Supplementary file 1 — Table S1 Summary of non‐cardiac findings on fetal magnetic resonance imaging (MRI) and additional postnatal or autopsy findings in patients with prenatal diagnosis of heterotaxy, sorted by left atrial isomerism (LAI; n = 19) or right atrial isomerism (RAI; n = 8), according to gestational week of fetal MRI investigation [file UOG-58-837-s001.docx]

**Gestational Ultrasound Fetal MRI Fetal MRI Fetal MRI Fetal MRI Fetal MRI Additional or discordant**

**age at fetal (prenatal) postmortem MRI (PmMRI),**

**MRI Non-cardiac Brain/face/ Lungs Gastrointestinal Spleen Urinary/ autopsy (AUT), or**

**(weeks) anomalies skeletal tract/liver umbilical vein postnatal imaging (PNI)**

**LAI**

20+2 Stomach Liver midline, Non-diagnostic abnormal

(right) gallbladder midline, (low image quality) course UV

stomach right,

ALC

20+3 Stomach Liver midline, Asplenia abnormal

(right) stomach right, course UV

ALC

21+1 SUA Liver midline, Asplenia abnormal PNI: Skeletal dysplasia, stomach left, course UV malrotation, short rib

ALC polydactyly,

polysplenia

21+3 Stomach Ventricular Liver midline, Asplenia abnormal

26+6 (right) asymmetry stomach right course UV

21+4 Cleft lip Cleft lip palate, Liver midline, Abnormal AUT: Poly-/syndactyly,

palate, short hypertelorism, stomach right spleen gallbladder aplasia, short

femur short curved femur intestines, small spleen bilobular lungs bilateral

23+0 Dandy- Dandy- Liver midline, Spleen abnormal PmMRI: Omphalocele

Walker- Walker- gallbladder midline, (right) course UV AUT: bilobular lungs

Malformation Malformation stomach right, bilateral

malrotation,

ALC

24+1 SUA, growth Situs inversus abd., Asplenia PNI: Polysplenia

restriction ALC

24+2 SUA, Bronchial tree Situs inversus abd. Spleen (right)

31+2 polyhydramnios anomalous

24+3 Stomach Bronchial tree Liver midline, Asplenia PNI: Polysplenia

(right) anomalous stomach right,

ALC

24+4 Double bubble Liver midline Polysplenia PNI: duodenal atresia not

gallbladder midline, confirmed

stomach right

duodenal atresia, malrotation

ALC

25+0 SUA, stomach Bronchial tree Liver midline, Asplenia

(right) anomalous stomach right

malrotation

ALC

26+4 Stomach Liver midline, Spleen

(right) gallbladder left, (right)

stomach right

26+4 Liver midline, Spleen Polycystic PNI: Polydactyly

stomach right, (right) kidneys

ALC

28+6 Bronchial tree Liver midline, Polysplenia

anomalous gallbladder left,

stomach right

malrotation

29+0 Stomach Liver midline, Abnormal PNI: Polysplenia

(right) gallbladder midline, spleen

stomach right,

duodenal atresia,

malrotation,

ALC

33+0 Bronchial tree Liver midline, Asplenia Duplex kidney PNI: Polysplenia,

anomalous stomach right, (right)

Malrotation,

ALC

33+2 Stomach Liver midline, Asplenia abnormal course PNI: malrotation,

(right), growth gallbladder aplasia, UV biliary atresia

restriction stomach right

36+2 Craniofacial Bronchial tree Liver midline, Spleen PRUV PNI: Asplenia,

dysmorphism anomalous stomach right, suspected hydronephrosis left ALC

37+0 Nutmeg Liver midline, Abnormal PNI: Asplenia

pattern stomach right spleen

**RAI**

17+6 SUA, Cerebellar Liver midline, Asplenia Kidney agenesis PmMRI: Rhombencephalo-

cerebellar hypoplasia, stomach right (right) synapsis, thymal agenesis hypoplasia, hydrocephalus, lung hypoplasia

hydrocephalus stenosis aqueduct

AUT: uterine agenesis,

dysplasia right thumb, monolobular right lung, trilobular left lung, scoliosis

21+6 Ventricular Liver midline, Asplenia PNI: Duodenal atresia, cleft

asymmetry gallbladder left, palate

stomach midline,

esophageal atresia

22+3 Stomach Bronchial tree Situs inversus abd., Spleen

(right) anomalous (right)

23+1 NT 2.9 mm Situs inversus abd. Spleen

(right)

23+2 Stomach Nutmeg Liver midline, Asplenia Duplex kidney AUT: trilobular lung bilateral

(right) pattern gallbladder midline, (right) bilateral

stomach right,

malrotation

25+5 SUA Bronchial tree Liver midline, Asplenia

anomalous gallbladder midline,

stomach left,

32+3 Ventriculo- Retrognathia, Nutmeg Liver midline, Asplenia abnormal course

megaly ventriculo- pattern stomach right, UV

megaly malrotation,

34+6 Bronchial tree Liver midline, Asplenia PNI: Hydrocephalus

anomalous gallbladder midline,

stomach left,

malrotation

ALC, abnormal liver configuration; AUT, autopsy; NT, nuchal translucency; PmMRI, post-mortem magnetic resonance imaging; PNI, postnatal imaging, PRUV, persistent right umbilical vein; SUA, single umbilical artery; UV, umbilical vein; Postnatal or postmortem findings in the last column are only listed if there was a discordance or a finding that had been missed prenatally. In all other cases prenatal findings were confirmed (but were not listed in this last column to facilitate reading)
